# Tunable Negative Thermal Expansion in Fe/Cr‐Substituted Nd_2_Co_17_ Compounds via Magnetoelastic Coupling

**DOI:** 10.1002/advs.202523129

**Published:** 2026-01-23

**Authors:** Jiayuan Li, Chenfei Qv, Haoran Tu, Qinfen Gu, Wayne D. Hutchison, Stewart J. Campbell, Zhenxiang Cheng, Wenquan Wang, Jianli Wang

**Affiliations:** ^1^ Center for Neutron Scattering and Advanced Light Source Science and Technology Dongguan University of Technology Dongguan P. R. China; ^2^ College of Physics Jilin University Changchun P. R. China; ^3^ Australian Synchrotron ANSTO Clayton Victoria Australia; ^4^ School of Science UNSW Canberra at the Australian Defence Force Academy Canberra Australian Capital Territory Australia; ^5^ Institute for Superconductivity and Electronic Materials University of Wollongong Wollongong New South Wales Australia

**Keywords:** controllable thermal expansion, magnetoelastic coupling, magnetic phase transition

## Abstract

Precisely tunable thermal expansion materials are essential for high‐precision applications, yet composition‐dependent bidirectional switching between positive and negative thermal expansion (NTE) poses a significant challenge. Here, we present a magnetoelastic approach to tailor anisotropic thermal expansion in Nd_2_(Co_1‐x_Fe_x_)_17‐y_Cr_y_ compounds. Synchrotron X‐ray diffraction studies reveal that increasing Fe content induces a reversible lattice response: while Co‐rich compositions (e.g., Nd_2_(Co_0.5_Fe_0.5_)_13.7_Cr_3.3_) display conventional positive expansion, Fe‐rich variants (e.g., Nd_2_(Co_0.2_Fe_0.8_)_14.7_Cr_2.3_) exhibit pronounced uniaxial NTE along the *c*‐axis (*α_c_* = −5.23 × 10^−6^ K^−1^) below Curie temperature (*T_C_*). This transition originates from strong magnetoelastic coupling at specific crystallographic sites, where enhanced occupancy at the 6c Wyckoff position strengthens negative exchange interactions, constraining the *c*‐axis via magnetostriction and inducing an anomalous thermal response with negative linear expansion. Compositional tuning not only suppresses the volume expansion coefficient (reducing *α_V_* by 20% at *x* = 0.7), but also induces a systematic, non‐monotonic modulation of *T_C_* (442–625 K). Critical exponent analyses near *T_C_* align with mean‐field theory, confirming the dominant role of long‐range magnetic interactions. Our findings establish a new strategy for achieving zero or near‐zero thermal expansion in rare‐earth intermetallic, making these materials promising candidates for precision thermal management.

## Graphical abstract

This study achieves anisotropic thermal expansion tuning in Nd_2_(Co_1‐x_Fe_x_)_17‐y_Cr_y_ compounds via a magnetoelastic strategy. Variable‐temperature synchrotron X‐ray diffraction reveals that increased Fe content induces switchable lattice responses. Compositional control reduces the volume expansion coefficient *α*V by 20% (x═0.7) and modulates T_C_ (442–625 K), establishing a novel pathway to zero/near‐zero thermal expansion in rare‐earth intermetallic.

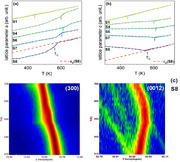

## 1 Introduction

The vast majority of materials exhibit thermal expansion, where their volume increases with rising temperature and contracts upon cooling – a phenomenon that often proves detrimental in precision applications, such as measurement equipment and laser instruments, where dimensional stability is paramount [1, 2, 3, 4, 5, 6]. To address this challenge, negative thermal expansion (NTE) materials have become a research focus. Recently, negative thermal expansion materials have achieved remarkable advances: high – entropy antiperovskite nitrides have extended the working temperature range of NTE to a span of 235 K (from 5 to 240 K), by leveraging the sluggish phase transition induced by entropy engineering [7]; Er‐Co‐Fe ferrimagnets (a typical rare‐earth transition‐metal system) have realized high‐performance zero thermal expansion (ZTE) with a volumetric coefficient of *α_V_* = 2.7 ppm K^−1^ over 10–220 K via sublattice‐magnetovolume effect [8]; and ultralight NaB(CN)_4_ has demonstrated giant NTE with a volume expansion coefficient of −9.2 × 10^−5^ K^−1^ (100–200 K) driven by low‐frequency phonon modes [9]. Despite these progresses, critical limitations persist for practical precision scenarios: most systems focus on bulk isotropic expansion regulation and lack the capability to tailor directional (anisotropic) expansion. Moreover, many systems only exhibit single‐mode NTE or ZTE behaviour without realizing continuously tunable thermal expansion, which poses significant challenges for achieving precise thermal management in sophisticated applications. Rare‐earth transition‐metal intermetallic compounds (R‐T; R is a rare earth and T is a transition metal) have emerged as promising candidates for thermal management due to their exceptional magnetic properties and minimal thermal expansion [10, 11, 12, 13]. In these materials, the rare‐earth sublattice contributes strong magnetic anisotropy, while the transition metal sublattice governs high Curie temperatures and spontaneous magnetization. Among them, the binary R_2_Fe_17_ system is particularly noteworthy, crystallizing in either the hexagonal Th_2_Ni_17_‐type structure (space group *P6_3_/mmc*) or the rhombohedral Th_2_Zn_17_‐type structure (space group *R‐3m*) depending on the rare‐earth element [14].

Controlling thermal expansion in functional materials remains a critical challenge. Alloying rare‐earth intermetallics with additional metal elements offers an effective strategy to tailor their crystal structures and magnetic properties, thereby fine‐tuning thermal expansion behaviour. As established by Givord and Lemaire [15], R_2_Fe_17_ compounds exhibit both positive and negative exchange interactions depending on Fe─Fe interatomic distances. Specifically, interactions between Fe atoms at *6c‐6c* and *9d‐18f* sites (distances < 2.45 Å) are antiferromagnetic, while longer distances yield ferromagnetic coupling. This competition between exchange interactions enables substantial manipulation of Curie temperatures (*T_C_*) and related magnetic properties through partial substitution of Fe by a third element T in R_2_Fe_17‐x_T_x_ compounds [16, 17]. In contrast, the absence of such competing interactions in R_2_Co_17_ analogues suggests fundamentally distinct responses to substitution in R_2_Co_17‐x_T_x_ systems [14, 17, 18].

In this work, we systematically investigate the structural and magnetic properties of Nd_2_(Co_1‐x_Fe_x_)_17‐y_Cr_y_ compounds using temperature‐dependent synchrotron X‐ray diffraction and magnetic measurements. Our study focuses on three interconnected phenomena: magnetic phase transitions, structural distortions, and thermal expansion behaviour. We demonstrate that progressive substitution of Cr and Fe for Co enables controlled switching from positive to negative anisotropic thermal expansion. These findings provide fundamental insights into magnetoelastic coupling mechanisms while advancing the design of materials with tailored thermal expansion properties for precision applications.

## 2 Results and Discussion

### 2.1 Structure Properties

**FIGURE 1:**
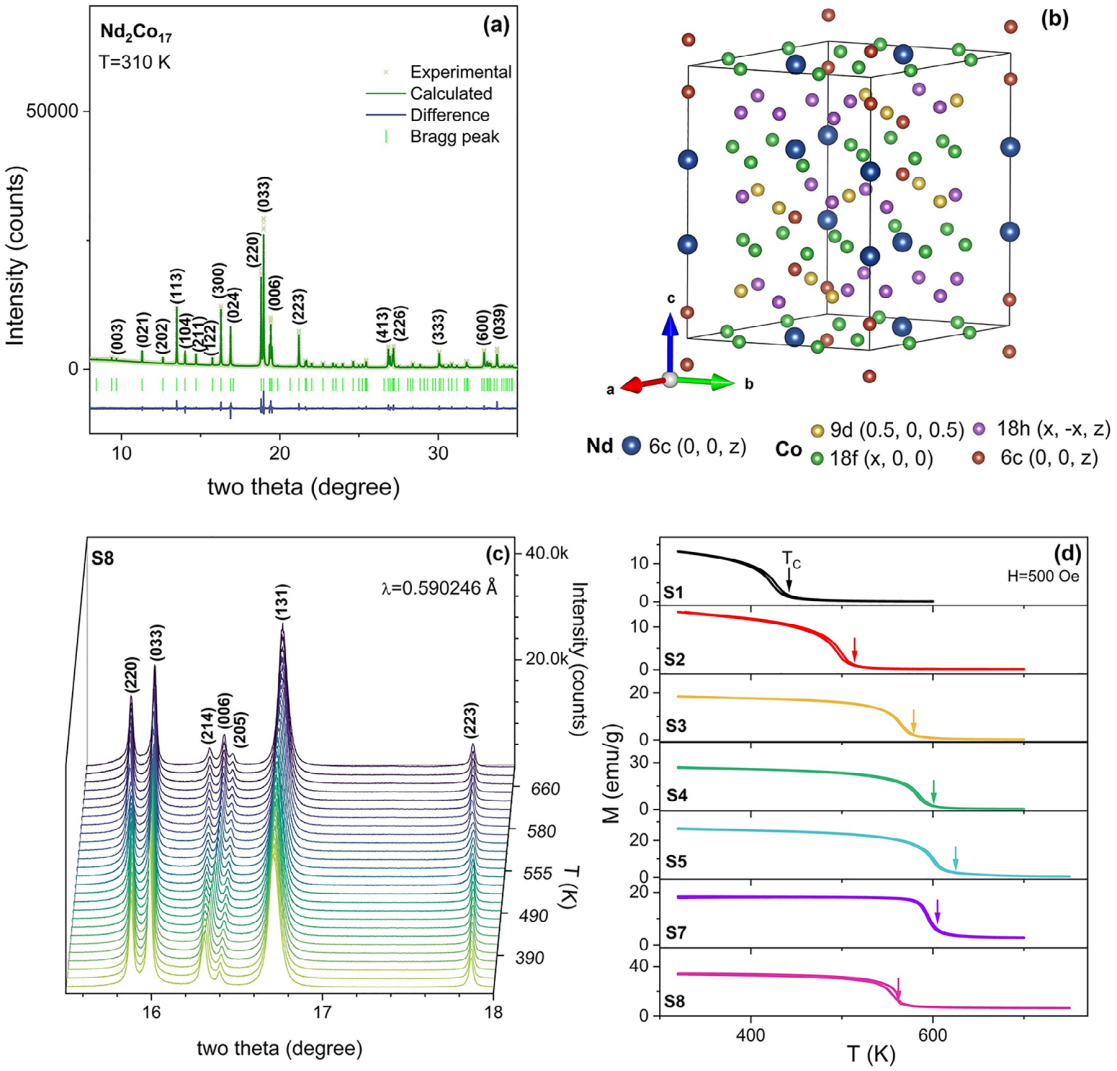
(a) XRD diffraction patterns and Rietveld refinement of the Nd_2_Co_17_ sample at *T* = 310 K. The experimental data are marked with hollow circles. The dark green line represents the Rietveld refined structure (using Fullprof) of the data. The light green vertical lines indicate the Bragg reflections, and the blue horizontal line shows the difference between the experimental and calculated patterns; (b) Crystal structure of Nd_2_Co_17_ (rhombohedral Th_2_Zn_17_‐type crystal structure); (c) Synchrotron radiation X‐ray diffraction patterns of the Nd_2_(Co_0.2_Fe_0.8_)_14.7_Cr_2.3_ sample (sample S8) in the temperature range of 310 – 700 K; (d) Temperature dependence of the magnetization for the Nd_2_(Co_1‐x_Fe_x_)_17‐y_Cr_y_ samples with *x* = 0.1, *y* = 4.7 (S1); *x* = 0.2, *y* = 4.3 (S2); *x* = 0.3, *y* = 4.0 (S3); *x* = 0.4, *y* = 3.7 (S4); *x* = 0.5, *y* = 3.3 (S5); *x* = 0.7, *y* = 2.7 (S7); *x* = 0.8, *y* = 2.3 (S8) in a field of *H* = 500 Oe. The *T_C_* values as determined by measurements of *M^2^(T)* versus *T* (Figure S2) are indicated by arrows.

To facilitate clarity in correlating sample compositions with experimental results, the sample codes and their corresponding complete chemical formulas are summarized in Table 1. Rietveld refinement of X‐ray diffraction patterns for Nd_2_Co_17_ at 310 K (Figure 1a) confirms that all samples are predominantly single‐phase and adopt the expected rhombohedral Th_2_Zn_17_‐type structure (space group: *R‐3m*) [14]. As illustrated in Figure 1b, the crystal structure contains one Wyckoff site for rare‐earth atoms (6c site (0, 0, z)) and four distinct Wyckoff sites for transition‐metal atoms: (6c site (0, 0, z), 9d site (0.5, 0, 0.5), 18f site (x, 0, 0), 18h site (x, ‐x, z)) [14].

**TABLE 1:** Sample codes, compositional parameters (x, y values), and complete chemical formulae of the Nd_2_(Co_1‐x_Fe_x_)_17‐y_Cr_y_ compounds.

Sample Code	Nd_2_(Co_1‐x_Fe_x_)_17‐y_Cr_y_		Complete Chemical Formula
	x Value	y Value	
S1	0.1	4.7	Nd_2_(Co_0.9_Fe_0.1_)_12.3_Cr_4.7_
S2	0.2	4.3	Nd_2_(Co_0.8_Fe_0.2_)_12.7_Cr_4.3_
S3	0.3	4.0	Nd_2_(Co_0.7_Fe_0.3_)_13_Cr_4_
S4	0.4	3.7	Nd_2_(Co_0.6_Fe_0.4_)_13.3_Cr_3.7_
S5	0.5	3.3	Nd_2_(Co_0.5_Fe_0.5_)_13.7_Cr_3.3_
S6	0.6	3.0	Nd_2_(Co_0.4_Fe_0.6_)_14_Cr_3_
S7	0.7	2.7	Nd_2_(Co_0.3_Fe_0.7_)_14.3_Cr_2.7_
S8	0.8	2.3	Nd_2_(Co_0.2_Fe_0.8_)_14.7_Cr_2.3_

Variable‐temperature synchrotron X‐ray diffraction was performed from 310 to 1040 K. As shown in Figure 1c for Nd_2_(Co_0.2_Fe_0.8_)_14.7_Cr_2.3_ (Sample S8), the crystal structure remains rhombohedral Th_2_Zn_17_‐type across the entire temperature range. However, splitting and shifting of the (006) peak suggest temperature‐dependent changes in certain lattice parameters.

In the *R‐3m* structure, variations in lattice parameters *a* and *c* near *T_C_* can be tracked using the (300) reflection (representing the ab‐plane) and the (0012) reflection (representing the *c*‐axis) [19]. As shown in Figures S1(a)–(d), the (300) peaks of all four compositions shift to lower angles with increasing temperature, indicating positive thermal expansion in the *ab*‐plane. In contrast, the (0012) peaks exhibit distinct anomalies. For Nd_2_(Co_0.5_Fe_0.5_)_13.7_Cr_3.3_ (sample S5) (Figure S1(a)), the (0012) peak shows a slight inflection near *T_C_* but continues shifting to lower angles, consistent with positive thermal expansion (*|Δ2θ|* ∼ 0.03066 for *ΔT* ∼ 320 K). With higher Fe content in Nd_2_(Co_0.4_Fe_0.6_)_14_Cr_3_ (sample S6) (Figure S1(b)), the rate of peak shift decreases significantly (*|Δ2θ|* ∼ 0.00987 for *ΔT* ∼ 305 K). For Nd_2_(Co_0.3_Fe_0.7_)_14.3_Cr_2.7_ (sample S7) and Nd_2_(Co_0.2_Fe_0.8_)_14.7_Cr_2.3_ (sample S8) (Figure S1(c,d)), the (0012) peak shifts toward higher angles below *T_C_* (∼ 605 and ∼ 562 K, respectively), indicating negative thermal expansion along the *c*‐axis. Above *T_C_*, the behaviour reverts to positive thermal expansion.

Temperature‐dependent magnetization measurements performed under *H* = 500 Oe (Figure 1d) reveal that all Nd_2_(Co_1‐x_Fe_x_)_17‐y_Cr_y_ compounds exhibit ferromagnetic behaviour, with a clear phase transition at the Curie temperature *T_C_*. Values of *T_C_* were determined by linearly extrapolating the *M^2^(T)* versus *T* curve to *M* = 0 (Figure S2) and are summarized in Table S1 [20]. Although Fe and Cr substitution does not alter the crystal structure, it leads to noticeable variations in lattice parameters and Curie temperatures *T_C_* (Table S1).

The observed non‐monotonic variation of *T_C_* in Nd_2_(Co_1‐x_Fe_x_)_17‐y_Cr_y_ can be understood by considering the interplay of two doping effects. First, the introduction of Fe brings into the system the characteristic distance‐dependent competition of Fe─Fe exchange, which can be ferromagnetic or antiferromagnetic depending on the interatomic distance. Second, Cr substitutes with a distinct site preference. At lower doping levels, Cr atoms preferentially occupy sites that mitigate the competing antiferromagnetic interactions among the newly introduced Fe atoms, thereby enhancing the net ferromagnetic coupling and causing *T_C_* to rise. However, with further Cr substitution, its role shifts: the dominant effect becomes a general dilution of the magnetic lattice and a weakening of essential ferromagnetic exchange pathways, leading to the eventual decrease in *T_C_*. This mechanism – where Fe doping establishes a tunable, competitive exchange landscape and Cr doping selectively modifies its balance – explains the non‐monotonic trend. It is consistent with the *T_C_* behaviour reported for other R–T systems where the composition modulates competing interactions, such as R_3_(Fe, Co, Cr)_29_ (R = Sm, Gd) [21, 22]. This stands in contrast to the monotonic decrease in *T*_C_ seen in parent R_2_Co_17_ upon doping with Cr or Fe, where such a sensitive competitive framework is absent [14, 18, 23].

### 2.2 Thermal Expansion

**FIGURE 2:**
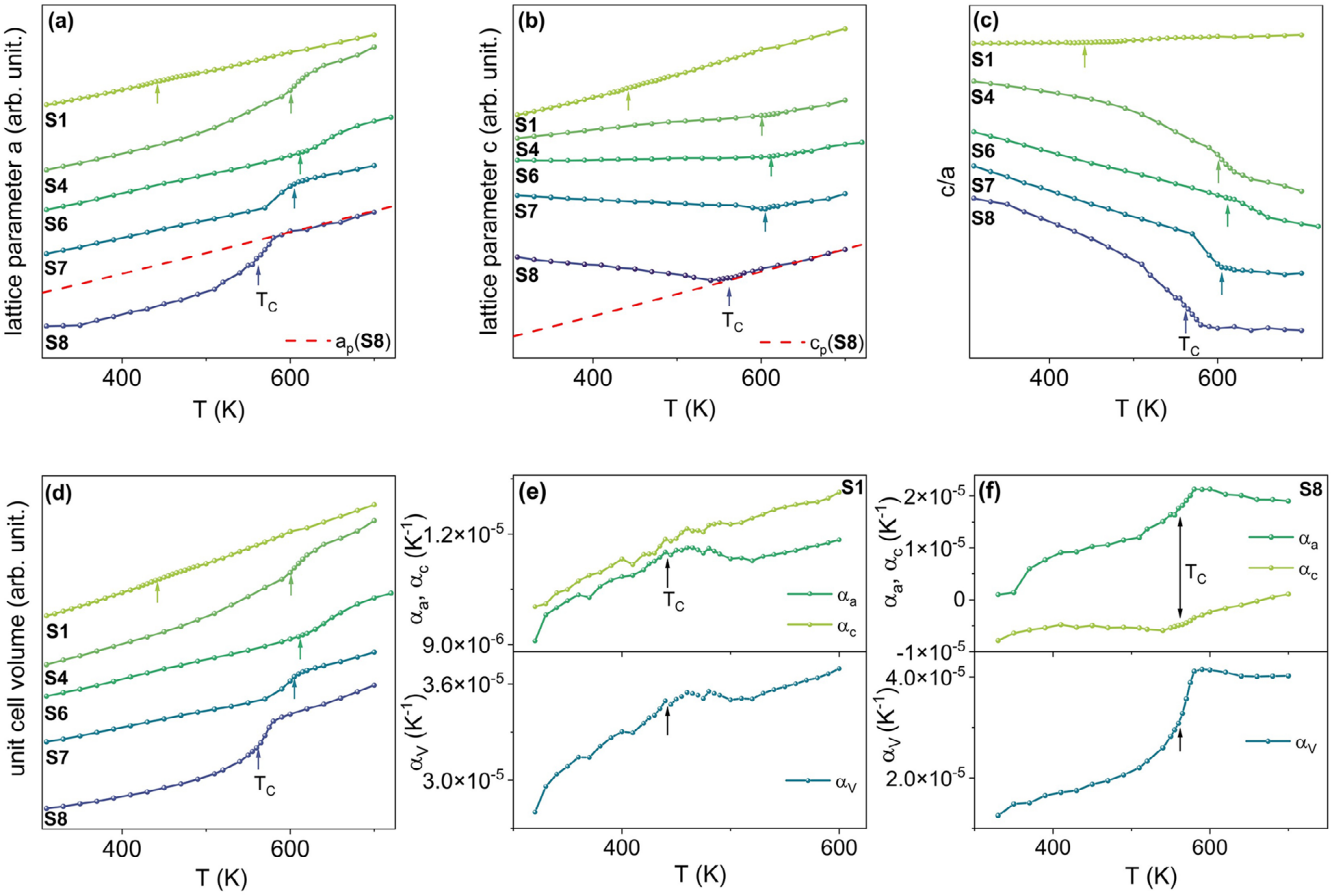
The temperature dependence of (a) lattice parameter *a*, (b) lattice parameter *c*, (c) *c/a* ratio, and (d) unit cell volume *V* for the samples Nd_2_(Co_0.9_Fe_0.1_)_12.3_Cr_4.7_ (sample S1), Nd_2_(Co_0.6_Fe_0.4_)_13.3_Cr_3.7_ (sample S4), Nd_2_(Co_0.4_Fe_0.6_)_14_Cr_3_ (sample S6), Nd_2_(Co_0.3_Fe_0.7_)_14.3_Cr_2.7_ (sample S7), Nd_2_(Co_0.2_Fe_0.8_)_14.7_Cr_2.3_ (sample S8), with Curie temperatures indicated by arrows. (The red dashed lines show the extrapolated paramagnetic lattice parameters (*a_p_*, *c_p_*)); (e) Temperature dependence of thermal expansion coefficients *α_a_*, *α_c_*, and *α_V_* for Nd_2_(Co_0.9_Fe_0.1_)_12.3_Cr_4.7_ sample (sample S1), and (f) Nd_2_(Co_0.2_Fe_0.8_)_14.7_Cr_2.3_ sample (sample S8).

Temperature‐dependent lattice parameters *a* and *c*, and unit cell volume *V* for Nd_2_(Co_0.9_Fe_0.1_)_12.3_Cr_4.7_ (sample S1), Nd_2_(Co_0.6_Fe_0.4_)_13.3_Cr_3.7_ (sample S4), Nd_2_(Co_0.4_Fe_0.6_)_14_Cr_3_ (sample S6), Nd_2_(Co_0.3_Fe_0.7_)_14.3_Cr_2.7_ (sample S7), and Nd_2_(Co_0.2_Fe_0.8_)_14.7_Cr_2.3_ (sample S8) were determined from variable‐temperature synchrotron data via Rietveld refinement. As shown in Figure 2a–d, all samples except Nd_2_(Co_0.9_Fe_0.1_)_12.3_Cr_4.7_ (sample S1) exhibit clear inflection points in *a*, *c*, and *V* near their respective Curie temperatures. Notably, increasing Fe content induces a systematic transition in the thermal expansion behaviour of the *c*‐axis below *T_C_*: from positive thermal expansion (PTE) in low‐Fe compounds to pronounced negative thermal expansion (NTE) in Fe‐rich ones. Specifically, low‐Fe compositions (Nd_2_(Co_0.9_Fe_0.1_)_12.3_Cr_4.7_ (sample S1) and Nd_2_(Co_0.6_Fe_0.4_)_13.3_Cr_3.7_ (sample S4)) show a gradual increase in *c* with temperature below *T_C_*, whereas the intermediate composition Nd_2_(Co_0.4_Fe_0.6_)_14_Cr_3_ (sample S6) exhibits nearly zero thermal expansion along *c* (Figure 2b). In contrast, high‐Fe compounds Nd_2_(Co_0.3_Fe_0.7_)_14.3_Cr_2.7_ (sample S7) and Nd_2_(Co_0.2_Fe_0.8_)_14.7_Cr_2.3_ (sample S8) display unambiguous NTE, with *c* decreasing as temperature rises from 310 K up to their respective *T_C_* values of 605 and 562 K. Detailed numerical values of *c* over the measured temperature range are provided in Table S1 in Section S1.

To further probe the thermal expansion behaviour and magneto‐structural coupling in Nd_2_(Co_1‐x_Fe_x_)_17‐y_Cr_y_, Figure S5(a)–(h) compare the temperature evolution of the experimental lattice parameters (*a_m_*, *c_m_*) and unit cell volume (*V_m_*) with extrapolated paramagnetic values (*a_p_*, *c_p_*, *V_p_*) shown as red lines. Above *T_C_*, in the stable paramagnetic regime (slightly above *T_C_* to avoid precursor effects), the thermal expansion follows the Debye model [14].

(1) $$C_{V}\;\left(\frac{T}{T_{D}}\right)=9R{\left(\frac{T}{T_{D}}\right)}^{3}\int_{0}^{\frac{T_{D}}{T}}\xi^{4}e^{\xi }/{\left(e^{\xi }-1\right)}^{2}d\xi$$

where *C_V_* is the specific heat, *T_D_* is the Debye temperature, and *R* is the molar gas constant. This model applies in this regime due to harmonic lattice vibrations and negligible magnetic interactions. The Debye temperature *T_D_* = 450 K used in the Debye model for the paramagnetic thermal expansion extrapolation is adopted from the literature on isostructural R_2_Fe_17_ compounds, where it was determined from acoustic measurements [24]. Given the structural and bonding similarities, this value serves as a reasonable approximation for our Nd_2_(Co_1‐x_Fe_x_)_17‐y_Cr_y_ system above *T_C_*. Below *T_C_*, however, the system enters a ferromagnetic state where strong magneto‐lattice coupling breaks the harmonic approximation. With increasing Fe content, Fe atoms preferentially occupy the 6c sites, and their ordered magnetic moments induce non‐harmonic lattice distortions. To relieve the effect of magnetostrain, the interatomic spacing along the *6c‐6c* bonds (parallel to the *c*‐axis) adjusts, causing deviations between *c_m_* and *c_p_*. In high‐Fe compounds such as Nd_2_(Co_0.3_Fe_0.7_)_14.3_Cr_2.7_ (sample S7) [Figure S5(g)] and Nd_2_(Co_0.2_Fe_0.8_)_14.7_Cr_2.3_ (sample S8) (Figure S5(h)), *c_m_* decreases with increasing temperature below *T_C –_* a behaviour incompatible with the Debye model. This magnetostrain‐driven lattice adjustment results in NTE below *T_C_*, clearly evidenced by the divergence between experimental and paramagnetic‐extrapolated values.

The thermal expansion coefficients were calculated using:

(2) $$\alpha_{l}=\frac{\Delta L}{\Delta T\times L_{0}}\;$$

(3) $$\alpha_{V}=\frac{\Delta V}{V_{0}\times \Delta T}\;$$

where *ΔL* = *L_1_*_–_ *L_0_* and *ΔV* = *V_1_*_–_ *V_0_* represent the length and volume changes over the temperature range *ΔT* = *T_1_* – *T_0_*. The temperature‐dependent linear expansion coefficients *α_a_, α_c_*, and volume coefficient *α_V_* for Nd_2_(Co_0.9_Fe_0.1_)_12.3_Cr_4.7_ (sample S1), Nd_2_(Co_0.5_Fe_0.5_)_13.7_Cr_3.3_ (sample S5), Nd_2_(Co_0.4_Fe_0.6_)_14_Cr_3_ (sample S6), and Nd_2_(Co_0.3_Fe_0.7_)_14.3_Cr_2.7_ (sample S7), and Nd_2_(Co_0.2_Fe_0.8_)_14.7_Cr_2.3_ (sample S8) are shown in Figure 2e–f and Figure S6(a)–(c). All five samples show inflection points in *α_a_(T), α_c_(T), and α_V_(T)* near their respective *T_C_*. Most strikingly, the *c*‐axis thermal expansion coefficient *α_c_* evolves systematically with Fe content: from positive through near‐zero to distinctly negative. In temperature intervals where *α_c_* remains nearly constant (fluctuation < ± 0.6 × 10^−6^ K^−1^), the average values reveal a clear progression: Nd_2_(Co_0.5_Fe_0.5_)_13.7_Cr_3.3_ (sample S5) shows PTE of 2.85 × 10^−6^ K^−1^ between 470–640 K; for Fe content *x* = 0.6, *α_c_* drops to 5.93 × 10^−7^ K^−1^ over 450–600 K (near‐zero thermal expansion, NZTE); further increasing the Fe content to *x*  = 0.7 yields NTE of −2.42 × 10^−6^ K^−1^ (410–570 K); and Nd_2_(Co_0.2_Fe_0.8_)_14.7_Cr_2.3_ (sample S8) exhibits enhanced NTE of −5.23 × 10^−6^ K^−1^ from 410–565 K. This continuous PTE‐NTE transition underscores the highly tunable magnetoelastic coupling in these compounds. Moreover, compositional tuning not only tailors the *c*‐axis expansion but also suppresses the volume expansion coefficient *α_V_* by ∼20%, from 2.58 × 10^−5^ K^−1^ in Nd_2_(Co_0.5_Fe_0.5_)_13.7_Cr_3.3_ (sample S5) to 2.07 × 10^−5^ K^−1^ in Nd_2_(Co_0.3_Fe_0.7_)_14.3_Cr_2.7_ (sample S7). These results suggest that Nd_2_(Co_1‐x_Fe_x_)_17‐y_Cr_y_ compounds are promising as controllable thermal expansion materials. By precisely adjusting the Fe/Cr ratio – for example, fixing Fe content at 0.60 ± 0.05 to maintain |*α_c_* | <1 × 10^−6^ K^−1^ over a temperature range >150 K – zero or near‐zero thermal expansion along the *c*‐axis can be achieved within targeted temperature windows. Building on this work, further elemental optimization could extend this control to the volume expansion coefficient *α_V_*, enabling zero or near‐zero volumetric expansion – a critical feature for precision devices, optical systems, and microelectronics requiring exceptional dimensional stability. Given the strongly anisotropic, uniaxial nature of the NTE (confined to the *c*‐axis), future efforts could focus on two distinct pathways: exploiting the anisotropy, for instance by engineering composite or layered materials that use the *c*‐axis NTE to compensate for the positive expansion of other components in a specific direction; or conversely, mitigating the anisotropy through further chemical substitution (e.g., with Al, Ga, or Si) to tame the positive expansion along the *a*‐axis, thereby achieving true volumetric control in a single phase. This unique anisotropic property itself opens avenues for designing devices where targeted dimensional tuning along a particular crystal axis is desired.

Generally speaking, negative thermal expansion (NTE) arises from diverse mechanisms, which can be categorized into four main types: phase transition, phonons (low‐energy transverse vibrational modes), rigid unit modes, and magnetic transition [25]. Corresponding to these mechanisms, typical NTE materials include: (1) ΑΜ_2_Ο_8_‐series compounds (e.g., ZrW_2_O_8_) with NTE driven by rigid unit modes, exhibiting a linear thermal expansion coefficient *α_l_* = −9.07 × 10^−6^ K^−^^1^ (2 – 350 K) [26]; (2) phase transition‐induced NTE materials represented by PbTiO_3_ (*α_l_* = −3.3 × 10^−6^ K^−^^1^, 303 – 673 K); (3) Α_2_M_3_O_12_‐series compounds (e.g., Lu_2_W_3_O_12_) where NTE originates from the synergy of low‐energy transverse phonons and rigid unit modes (*α_l_* = −6.8 × 10^−6^ K^−^^1^) [2]; and (4) the R_2_Fe_17_‐series compounds investigated in this work, whose NTE effect is attributed to magnetoelastic coupling associated with magnetic phase transitions.

Compared to the mechanistic constraints of other NTE systems, the magnetoelastic mechanism in R_2_Fe_17_ compounds offers unique features: first, unlike the fixed rigid unit modes (ΑΜ_2_Ο_8_/Α_2_M_3_O_12_) or discontinuous phase transitions (PbTiO_3_), magnetoelastic coupling here is dynamically controllable via simple elemental doping – Fe/Cr substitution modulates the strength of spin‐lattice interactions across different Wyckoff sites (as discussed in detail in Section 2.3), enabling continuous tuning of thermal expansion from positive thermal expansion (PTE) to near‐zero thermal expansion (NZTE) and NTE, rather than being confined to a single expansion mode. Second, this magnetic‐driven mechanism avoids the thermal instability observed in some oxide‐based NTE materials (e.g., ZrW_2_O_8_’s metastable room‐temperature cubic phase and kinetic instability at temperatures above ∼1050 K) [27]. As intermetallic alloys, R_2_Fe_17_ compounds maintain structural integrity over a broad temperature range, while their synthesis (via conventional arc melting) is simpler and lower‐cost than the high‐pressure sintering required for oxides. Consequently, they represent promising candidates for controllable thermal expansion materials.

### 2.3 Magnetoelastic Coupling at Individual Sites

The crystal structure refinement of all samples was performed via Rietveld analysis of synchrotron XRD data using FullProf, following the structural constraints inherent to the Th_2_Zn_17_‐type structure (space group R‐3m) phase. To avoid unphysical overfitting and compensating for the inability of synchrotron XRD to differentiate Fe/Co/Cr occupancy, we mainly adopted two methods: First, the occupancy (Occ) of all transition‐metal sites (9d, 18f, 18h, 6c) was fixed to the stoichiometric values corresponding to the 2:17‐type structure (e.g., 0.25000 for 9d site, 0.50000 for18f/18h site, 0.16667 for 6c site) throughout the refinements. This constraint ensures the total number of transition‐metal atoms is consistent with the nominal chemical composition of the Th_2_Zn_17_‐type structure, adhering to the fundamental principle of stoichiometric conservation for intermetallic compounds. Consequently, by focusing on optimizing the atomic positional parameters (including z_6c_ for Nd, and x_18f_, x_18h_, z_18h_, and z_6c_ for Co/Fe/Cr atoms) – a parameter set for which synchrotron XRD exhibits high sensitivity – refinement provides reliable, fitting quality. Second, previous studies about ab initio‐derived interatomic potential‐based calculations on Nd_2_Co_17‐x_T_x_ (T = Fe or Cr) confirm Fe/Cr preferentially occupy the 6c site (maximizing system energy reduction), with a clear preference order of 6c > 18f > 18h > 9d [28]. This trend is corroborated by neutron diffraction studies on isostructural R_2_Fe_17_‐series compounds, which experimentally validate the same 6c‐prioritized occupancy sequence [14, 29, 30].

The pronounced magnetoelastic anomalies we observe – specifically the suppression of the 6c‐6c distance and the inflection in the *c*‐axis lattice parameter below *T_C_* in Fe‐rich composition Nd_2_(Co_0.2_Fe_0.8_)_14.7_Cr_2.3_ (sample S8) (Figure S7) – are fully consistent with and indicative of a high Fe/Cr presence at this pivotal 6c site. This site‐specific structural response would be unlikely if Fe/Cr occupied other positions less influential on *c*‐axis coupling. Thus, the observed behaviour and the established occupancy model are mutually reinforcing, providing a coherent explanation for the strong, tunable magnetoelastic coupling in this system.

Based on the established occupancy model, we next probe the local atomic environments to unravel the underlying magnetoelastic coupling mechanism. As illustrated in Figure 1b, Nd atoms in the Nd_2_(Co_1‐x_Fe_x_)_17‐y_Cr_y_ compounds occupy the 6c rare‐earth Wyckoff site, while Co, Fe, and Cr atoms are distributed across four transition‐metal Wyckoff positions: 9d, 18f, 18h, and 6c. Taking Nd_2_(Co_0.8_Fe_0.2_)_12.7_Cr_4.3_ (sample S2) as a representative example, coordination analysis shows that each Nd atom at 6c is surrounded by 19 near‐neighbor Co atoms – distributed as 3 atoms at 9d, 6 at 18f, 9 at 18h, and 1 at 6c – along with one adjacent Nd atom. At 310 K, the average Nd─Co bond length is 3.13 Å (±  6.76  ×  10^−3^ Å), as summarized in Table S2.

Transition‐metal atoms exhibit distinct coordination environments: those at 9d have 2 Nd and 10 Co neighbors (with 4 Co atoms located at 18f, 4 at 18h, 2 at 6c); at 18f, each atom is coordinated by 2 Nd and 11 Co atoms (2 at 9d, 3 at 18f, 4 at 18h, 2 at 6c); at 18h, the coordination includes 3 Nd and 9 Co atoms (2 at 9d, 2 at 18f, 4 at 18h, 1 at 6c); and at 6c, each atom bonds to 1 Nd and 13 Co atoms (3 at 9d, 6 at 18f, 3 at 18h, 1 at 6c). The average bond lengths between Co/Fe/Cr atoms at the 9d, 18f, 18h, and 6c sites and their neighboring Co atoms are 2.46, 2.64, 2.53, and 2.63 Å, respectively (Table S2). Using the atomic radii of Nd (1.82 Å) and Co/Fe/Cr (1.25 Å), Wigner–Seitz cell (WSC) volumes were computed via the BLOKJE program [31]. The resulting volumes are 31.00 Å^3^ for Nd at 6c, and 10.86, 11.25, 11.54, and 11.89 Å^3^ for Co/Fe/Cr at 9d, 18f, 18h, and 6c, respectively – values consistent with those reported for other R_2_Fe_17_‐type compounds [32, 33].

**FIGURE 3:**
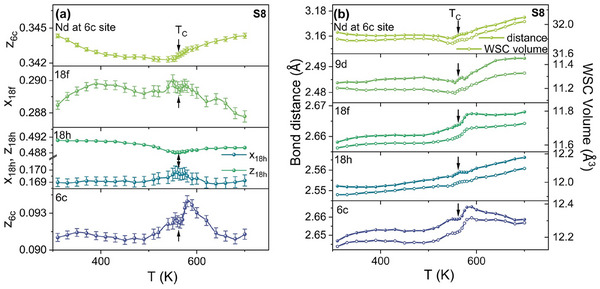
(a) Temperature dependence of atomic positional parameters z_6c_, x_18f_, x_18h_, z_18h_, and z_6c_ for the Nd_2_(Co_0.2_Fe_0.8_)_14.7_Cr_2.3_ sample (sample S8): Nd atoms at the 6c Wyckoff position and Co/Fe/Cr atoms at the 18f, 18h, and 6c Wyckoff positions; (b) Temperature dependence of average bonding distances between atoms at different Wyckoff positions and adjacent Co atoms, as well as Wigner–Seitz cell volumes, for the Nd_2_(Co_0.2_Fe_0.8_)_14.7_Cr_2.3_ sample (sample S8), with Curie temperatures indicated by arrows.

To probe the magnetoelastic coupling behaviour, we analyzed the temperature evolution of atomic position parameters: z_6c_ for Nd, and x_18f_, x_18h_, z_18h_, and z_6c_ for Co/Fe/Cr atoms. As shown in Figures S8(a)–(c) and Figure 3a, distinct inflections in these parameters occur near *T_C_* in Fe‐rich compositions, most notably in Nd_2_(Co_0.2_Fe_0.8_)_14.7_Cr_2.3_ (sample S8) (Figure 3a), thus signaling pronounced magnetoelastic coupling during the magnetic phase transition.

Further insight is gained from the temperature‐dependent WSC volumes and average bonding distances, also computed using BLOKJE. For the Fe‐rich compound Nd_2_(Co_0.2_Fe_0.8_)_14.7_Cr_2.3_ (sample S8), the key observation is that the average bond lengths across all Wyckoff sites exhibit clear inflection points near its Curie temperature, *T_C_* (Figure 3b). Specifically, although the bond lengths themselves remain largely constant within the experimental uncertainty (e.g., ∼2.48 Å for the 9d site and ∼2.55 Å for the 18h site), their temperature‐dependent evolution displays distinct, site‐specific deviations from monotonicity: a pronounced turnaround for the 9d site, a sharp change in slope for the 18f site, a subtle yet discernible slope variation for the 18h site, and a well‐defined inflection for the 6c site. The average Nd‐Co distance also shows a subtle decrease alongside a clear inflection. These inflection points, appearing synchronously near *T_C_*, serve as direct evidence of strong, site‐specific magnetoelastic coupling in Nd_2_(Co_0.2_Fe_0.8_)_14.7_Cr_2.3_ (sample S8). This behaviour stands in stark contrast to the monotonic thermal expansion observed in lower‐Fe analogues such as Nd_2_(Co_0.8_Fe_0.2_)_12.7_Cr_4.3_ (sample S2) (Figure S9(a)), and Nd_2_(Co_0.4_Fe_0.6_)_14_Cr_3_ (sample S6) (Figure S9(b)).

In summary, these site‐specific observations – including the suppressed 6c‐6c nearest‐neighbor distance, pronounced inflections in atomic position parameters near T_C_, and anomalous temperature‐dependent bonding distances across 6c, 9d, 18f, and 18h sites – unveil the atomistic origin of magnetoelastic coupling in Fe and Cr‐doped Nd_2_Co_17_ compounds. The stark contrast between the site‐selective structural anomalies in Fe‐rich compositions and the monotonic thermal expansion in low‐Fe analogues confirms that gradual Fe addition modulates magnetoelastic coupling strength at individual Wyckoff sites. This modulation directly governs the system's transition from positive to negative thermal expansion, providing a critical atomic‐scale rationale for the tunable thermal expansion behaviour of 2:17‐type rare‐earth transition‐metal intermetallics via Fe/Cr co‐substitution.

### 2.4 Magnetic Phase Transition

As is well known, two types of magnetic phase transitions may occur at the Curie point: first‐order magnetic transition (FOMT) and second‐order magnetic transition (SOMT). A FOMT is characterized by a discontinuous change in magnetization with latent heat, while a SOMT shows a continuous change in magnetization without latent heat. From the slope of an Arrott plot (graph of *M^2^* versus *H/M*), the order of the magnetic phase transition around the Curie temperature can be determined. Specifically, a negative slope indicates a first‐order magnetic transition, whereas a positive slope corresponds to a second‐order magnetic transition [34, 35, 36].

**FIGURE 4:**
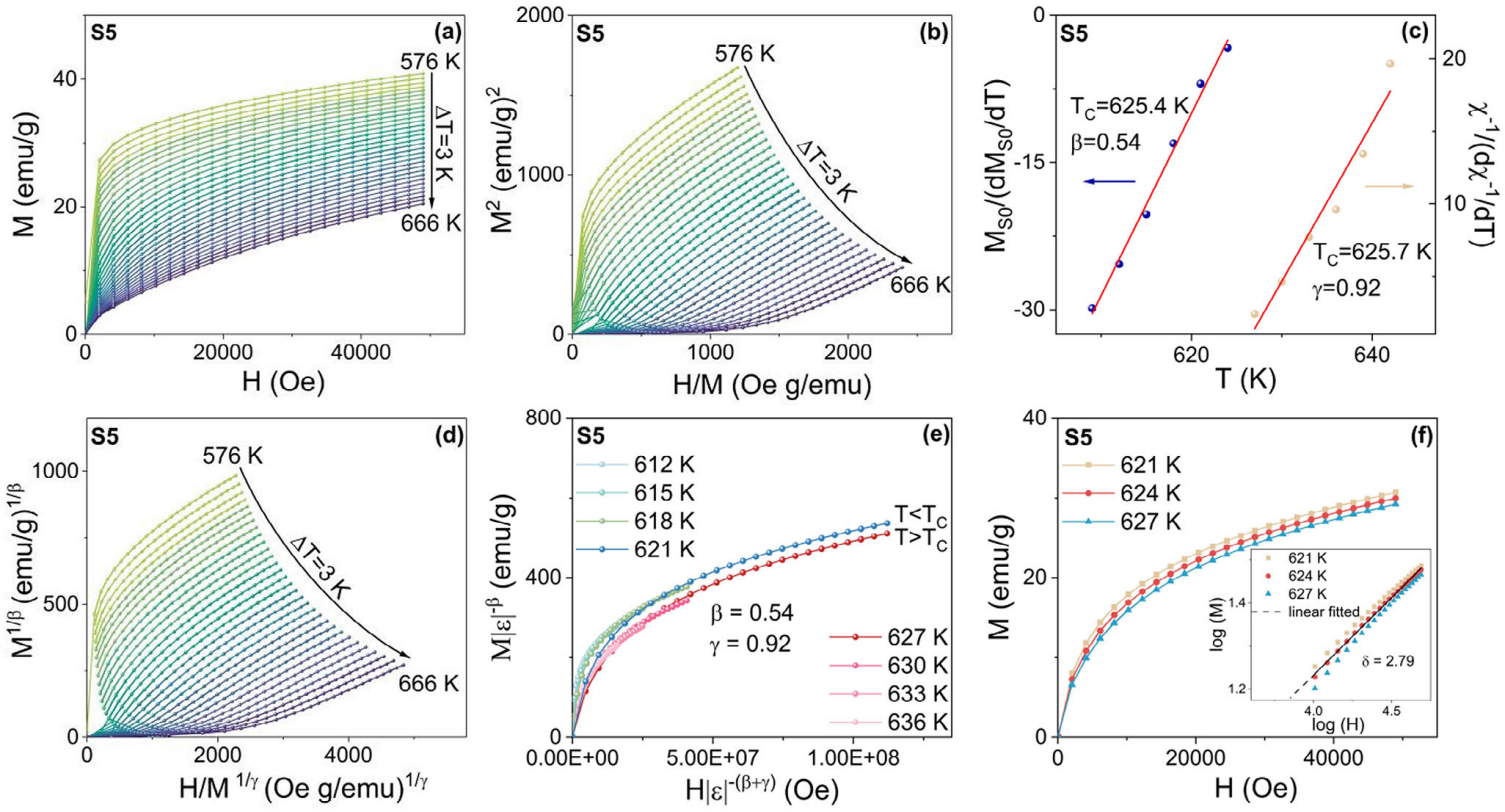
(a) Magnetization versus field curves (*H* = 0 – 50000 Oe) for the Nd_2_(Co_0.5_Fe_0.5_)_13.7_Cr_3.3_ sample (sample S5) around the Curie temperature; (b) Arrott plots of *M^2^* versus *H/M* for the S5 sample; (c) Kouvel‐Fisher plot for the spontaneous magnetization *M_S_(T)/(dM_S_/dT)* (left scale) versus temperature *T* and the inverse initial susceptibility *χ^−1^/(dχ^−1^/dT)* (right scale) versus temperature *T*; (d) Modified Arrott plots of *M^1/β^* as a function of *(H/M)^1/γ^* leading to the values *γ* = 0.92 and *β* = 0.54; (e) Scaling plots of *M|ε|^−β^* versus *H|ε|^−(β+γ)^* (using *β* and *γ* as discussed in the text; cf. Equation (9)), indicating the universal behaviour of the curves below and above *T_C_* for the S5 sample; (f) Critical isotherms of magnetization as a function of the magnetic field close to the Curie temperature for the S5 sample – the inset shows the data plotted on a log *M* versus log *H* graph and the linear fit obtained at the critical transition temperature.

Nd_2_(Co_0.5_Fe_0.5_)_13.7_Cr_3.3_ (sample S5) has been used as an example in order to investigate the type of magnetic phase transition that occurs at the Curie temperature in Nd_2_(Co_1‐x_Fe_x_)_17‐y_Cr_y_ compounds. As shown in Figure 4a, isothermal magnetization data for magnetic fields in the range *H* = 0 − 50 kOe have been measured at temperatures around the Curie temperature *T_C_* ∼ 625 K (Table S1). The temperature step between successive isothermal *M(H)* curves was 3 K. The Arrott plots derived from the magnetization data of this compound are presented in Figure 4b. The curves of *M^2^* versus *H/M* indicate that the Nd_2_(Co_0.5_Fe_0.5_)_13.7_Cr_3.3_ (sample S5) compound undergoes a second‐order magnetic phase transition near the Curie temperature.

To comprehensively understand the range of fundamental magnetic interactions, we conducted a detailed analysis of the critical behaviour of the magnetic phase transition around *T_C_*. According to the scaling hypothesis, the second‐order phase transition near the Curie temperature is characterized by a set of interdependent critical exponents *β*, *γ*, *δ*, and a magnetic equation of state. As functions of the reduced temperature $\varepsilon =\frac{T-T_{C}}{T_{C}}\;$, the spontaneous magnetization and initial susceptibility can be expressed as follows [37]:

(4) $$M_{S}\;\left(T\right)=M_{0}\;{\left(-\varepsilon \right)}^{\beta },\varepsilon <0,below\;T_{C}$$

(5) $$\chi_{0}^{-1}\;\left(T\right)=\left(\frac{h_{0}}{M_{0}}\right)\;\varepsilon^{\gamma },\varepsilon >0,above\;T_{C}$$

(6) $$M=DH^{1/\delta },\varepsilon =0,T=T_{C}\;$$

where *M_0_*, *h_0_/M_0_*, and *D* are the critical amplitudes. These phases are strictly valid only within a narrow range around *T_C_*. For this compound, *M_S_(T)* and *χ_0_^−1^(T)* can be calculated using the Arrott plots in Figure 4b, where *χ_0_^−1^(T)* and *M_S_^2^(T)* correspond to the positive values of the *H/M* intercept and the *M^2^* intercept, respectively. As previously confirmed in numerous systems, the Kouvel–Fisher method – utilizing Equations (7) and (8) presented below – represents a more precise approach for deriving the critical exponents *β* and *γ* [38]:

(7) $$\frac{M_{S}\left(T\right)}{\frac{dM_{S}\left(T\right)}{dT}}=\frac{T-T_{C}}{\beta }\;$$

(8) $$\frac{\chi_{0}^{-1}\left(T\right)}{\frac{d\chi_{0}^{-1}\left(T\right)}{dT}}=\frac{T-T_{C}}{\gamma }\;$$

Based on the above two formulae, plots of *M_S_(T)[dM_S_(T)/dT]^−1^* and *χ_0_^−1^(T)[dχ_0_^−1^(T)/dT]^−1^* versus temperature are expected to yield straight lines with slopes of 1/*β* and 1/*γ*, respectively, and the *T_C_* value obtained from the intercepts on the temperature axis. As shown in Figure 4c, good agreement is obtained for the Nd_2_(Co_0.5_Fe_0.5_)_13.7_Cr_3.3_ (sample S5) compound with exponents *β* and *γ*, as derived via the Kouvel–Fisher method, of *β* = 0.54 and *γ* = 0.92. In addition, the *T_C_* values obtained from the intercepts on the temperature axis – *T_C_* = 625.4 K (*β* intercept) and *T_C_* = 625.7 K (*γ* intercept) – are in very good agreement with the experimental value of *T_C_* = 625(±  5) K (Table S1).

Two key pieces of evidence validate the critical exponents *β* and *γ* obtained by the Kouvel–Fisher method: first, Figure 4d displays the modified Arrott plots of *M^1/β^* versus *(H/M)^1/γ^*. The clear parallelism of all lines in these plots directly validates that the critical exponents derived by the Kouvel–Fisher method match the data extremely well. Second, the scaling theory can also be utilized to validate the accuracy of the critical exponents *β* and *γ*. In the critical region, the magnetic equation of state is expressed as:

(9) $$M\;\left(H,\varepsilon \right)=\varepsilon^{\beta }\;f_{\pm }\left(\frac{H}{\varepsilon^{\beta +\gamma }}\right)$$

where *ε* is the reduced temperature, and *f_+_* and *f_−_* are regular analytical functions above and below *T_C_*, respectively. By substituting the *β* and *γ* values from the Kouvel–Fisher method, the scaled plots of *M|ε|^−β^* versus *H|ε|^−(β+γ)^* as in Figure 4e, yield two universal curves – one above and one below *T_C_* – in strict accordance with the scaling hypothesis. This result also confirms that both the critical exponents and the determined *T_C_* for this compound are reliable and consistent with theoretical predictions.

The critical component *δ* can be directly derived from the critical isotherm M using Equation (6). According to Equation (6), a plot of log(M) versus log(H) should yield a straight line with a slope of *1/δ*. As illustrated in the inset to Figure 4f, the *δ* value of the Nd_2_(Co_0.5_Fe_0.5_)_13.7_Cr_3.3_ (sample S5) compound, obtained from the critical isotherm at *T_C_* = 624 K, is *δ* = 2.79.

The critical behaviour of a system is categorized into distinct universality classes (UCs) according to the nature of spin‐spin interactions, such as Heisenberg; mean‐field; or Ising model featuring critical exponent values of: *β* = 0.365, *γ* = 1.386, *δ* = 4.80; *β* = 0.5, *γ* = 1, *δ* = 3; and *β* = 0.325, *γ* = 1.241, *δ* = 4.82, respectively [39]. Comparison of the present set of experimental values – *β* = 0.54, *γ* = 0.92, *δ* = 2.79 – with those for the distinct UCs, reveals that the critical exponents *β*, *γ*, and *δ* of the Nd_2_(Co_0.5_Fe_0.5_)_13.7_Cr_3.3_ (sample S5) compound closely resemble the theoretical values of the mean‐field interaction model dominated by long‐range interactions. Hence, it is concluded that the critical behaviour analysis at *T_C_* indicates that the magnetism of this set of Nd_2_(Co_1‐x_Fe_x_)_17‐y_Cr_y_ compounds is governed by long‐range interactions. In summary, the critical behaviour analysis establishes the long‐range nature of the magnetic order as the fundamental prerequisite for the strong magnetoelastic effects in this system. This finding provides a crucial microscopic foundation for the strong, composition‐tunable magnetoelastic coupling and the consequent thermal expansion anomalies observed and discussed in the preceding sections.

## 3 Conclusion

In summary, variable‐temperature synchrotron X‐ray diffraction analyses of Nd_2_(Co_1‐x_Fe_x_)_17‐y_Cr_y_ compounds reveal composition‐dependent thermal expansion behaviour dominated by magnetoelastic coupling. With increasing Fe content, the lattice parameter *c* undergoes a continuous transition from positive to negative thermal expansion below the Curie temperature, accompanied by a systematic reduction in the volume expansion coefficient. The overall nature of the thermal expansion behaviour in the ferromagnetic regions of the Nd_2_(Co_1‐x_Fe_x_)_17‐y_Cr_y_ system is indicated by the phase diagram of Figure S11 in which the various regions – PTE; PTNE to NZTE; NZTE to NTE and NTE – are shown as a function of Fe content. Critical exponent analysis near *T_C_* confirms that long‐range magnetic interactions govern the phase transition behaviour. These findings demonstrate that targeted compositional tuning – particularly through Fe/Cr ratio optimization – enables precise control over thermal expansion, making this family of rare‐earth intermetallics promising for applications requiring zero or near‐zero thermal expansion in precision instrumentation and thermal management systems.

## 4 Experimental Section

All samples of Nd_2_(Co_1‐x_Fe_x_)_17‐y_Cr_y_ (*x* = 0.0 – 0.8; *y* = 0.0 – 4.7) were prepared using the conventional arc melting method under an argon atmosphere. The raw materials, Nd, Fe, Co, and Cr, were at least 99.9% purity. Approximately 2% excess Nd was added to compensate for loss during melting and following the heat‐treatment process. To ensure the chemical homogeneity of samples, the ingots were reversed and remelted more than five times before being sealed in an evacuated quartz tube under high vacuum. The samples were then annealed at 900 °C for one week and finally quenched into water. Samples were characterized by X‐ray powder diffraction (XRD) measurements at room temperature using a PANAlytical diffractometer with Cu‐K_α_ radiation to evaluate the phase composition and crystal structure. Details of the quantitative elemental content for the Nd_2_(Co_0.3_Fe_0.7_)_14.3_Cr_2.7_ sample (S7) and the Nd_2_(Co_0.2_Fe_0.8_)_14.7_Cr_2.3_ sample (S8) are provided in Tables S3 and S4, showing good agreement with the nominal compositions. Similarly, the EDS spectrum and quantitative result for a Nd_2_Co_17_ sample is shown in Table S5. The synchrotron radiation x‐ray diffraction tests (*λ* = 0.590246 Å) were carried out over a temperature range of 303 – 1040 K at the powder diffraction beamline of the Australian Synchrotron in order to clarify the temperature dependence of the lattice parameters and to investigate the magnetoelastic coupling effects. The fine sample powders were loaded into the quartz capillary that was spun continuously during the collection of the synchrotron data. The temperature step was set as *ΔT* = 10 K near the Curie temperature *T_C_* and *ΔT* = 20 K in other temperature regions. Magnetization measurements were performed using a Quantum Design 14 T Physical Property Measurement System (PPMS) over the temperature range ∼ 310 K–750 K.

## Author Contributions

J.Y.L. contributed to the methodology, investigation, data curation, formal analysis, and preparation of the original draft. C.F.Q. was involved in the methodology, data curation, and formal analysis, while H.R.T. contributed to data curation and formal analysis. Q.F.G. and W.D.H. participated in the methodology and data curation. S.J.C. contributed to the formal analysis and was responsible for reviewing and editing the manuscript. Z.X.C. contributed to the methodology, data curation, and formal analysis, and also participated in manuscript review and editing and funding acquisition. W.Q.W. was responsible for the methodology, supervision, and manuscript review and editing. J.L.W. led the conceptualization and contributed to the methodology, investigation, data curation, and formal analysis, as well as supervision, validation, manuscript review and editing, and funding acquisition.

## Conflicts of Interest

The authors declare no conflicts of interest.

## Supporting information




**Supporting File**: advs73971‐sup‐0001‐SuppMat.docx.

## Data Availability

The data that support the findings of this study are available from the corresponding author upon reasonable request.
