# Supplementary material for: Tunable Negative Thermal Expansion in Fe/Cr‐Substituted Nd2Co17 Compounds via Magnetoelastic Coupling
Source: Adv Sci (Weinh). 2026 Jan 23:e23129. Online ahead of print. doi: 10.1002/advs.202523129 (PMC13325516; doi:10.1002/advs.202523129)
Supplement: Supplementary file 1 — Supporting File: advs73971‐sup‐0001‐SuppMat.docx. [file ADVS-9999-e23129-s001.docx]

**Tunable Negative Thermal Expansion in Fe/Cr-Substituted Nd_2_Co_17_ Compounds via Magnetoelastic Coupling**

*Jiayuan Li, Chenfei Qv, Haoran Tu^*^, Qinfen Gu, Wayne D. Hutchison, Stewart J. Campbell,* *Zhenxiang Cheng^*^,* *Wenquan Wang^*^ and* *Jianli Wang**

**Section 1: Temperature-dependent data of lattice parameter *c​***

As presented in **Figure S4**(b), for compound Nd_2_(Co_0.9_Fe_0.1_)_12.3_Cr_4.7_ (S1), the lattice parameter *c* shows a steady increase with rising temperature below its Curie temperature. Specifically, c increases from 12.268(5) Å at 310 K to 12.288(0) Å at 442 K (the Curie temperature of S1), corresponding to a positive thermal expansion with a total change of +0.019(5) Å over this temperature range.

Compound Nd_2_(Co_0.6_Fe_0.4_)_13.3_Cr_3.7_ (S4) exhibits a similar positive thermal expansion behaviour in the lattice parameter *c* below its Curie temperature. At 310 K, *c* is measured as 12.370(5) Å, and it gradually increases to 12.386(8) Å when the temperature reaches 601 K (the Curie temperature of S4), with a total increment of +0.016(3) Å, confirming the positive thermal expansion characteristic.

In the case of compound Nd_2_(Co_0.4_Fe_0.6_)_14_Cr_3_ (S6), the variation of lattice parameter *c* with temperature below its Curie temperature is relatively small. At 310 K, *c* is 12.430(1) Å, and it slightly changes to 12.432(9) Å at 612 K (the Curie temperature of S6), showing a negligible total change of +0.002(8) Å, indicating a nearly zero thermal expansion behaviour.

For compound Nd_2_(Co_0.3_Fe_0.7_)_14.3_Cr_2.7_ (S7), a distinct negative thermal expansion of the lattice parameter *c* is observed below its Curie temperature (605 K). The value of *c* decreases from 12.465(2) Å at 310 K to 12.455(4) Å at 605 K, with a total change of -0.009(8) Å as the temperature rises from 310 K to 605 K.

Compound Nd_2_(Co_0.2_Fe_0.8_)_14.7_Cr_2.3_ (S8) also displays a significant negative thermal expansion of lattice parameter *c* below its Curie temperature (around 562 K). At 310 K, *c* is 12.466(3) Å, and it decreases to 12.450(5) Å near 562 K, resulting in a total reduction of -0.015(8) Å over this temperature interval, further confirming the pronounced negative thermal expansion behaviour.

These detailed data clearly illustrate the transition in the thermal expansion behaviour of lattice parameter *c* from positive to negative with increasing Fe content, consistent with the observations in the main text.





**Figure S1.** Variation of (300) and (0012) peak positions with temperature for (a) Nd_2_(Co_0.5_Fe_0.5_)_13.7_Cr_3.3_ (S5), (b) Nd_2_(Co_0.4_Fe_0.6_)_14_Cr_3_ (S6), (c) & (e) Nd_2_(Co_0.3_Fe_0.7_)_14.3_Cr_2.7_ (S7), and (d) & (f) Nd_2_(Co_0.2_Fe_0.8_)_14.7_Cr_2.3_ samples (S8) (Curie temperatures indicated by arrow).





**Figure S2.** *M^2^(T)* versus *T* curve to *M* = 0 for the Nd_2_(Co_1-x_Fe_x_)_17-y_Cr_y_ samples with *x* = 0.1, *y* = 4.7 (S1); *x* = 0.2, *y* = 4.3 (S2); *x* = 0.3, *y* = 4.0 (S3); *x* = 0.4, *y* = 3.7 (S4); *x* = 0.5, *y* = 3.3 (S5); *x* = 0.7, *y* = 2.7 (S7); *x* = 0.8, *y* = 2.3 (S8) in a field of *H* = 500 Oe.





**Figure S3.** Synchrotron radiation X-ray diffraction patterns of the Nd_2_Co_17_ sample in the temperature range of 310 K - 1040 K.

**Table S1.** The Curie temperature of Compound Nd_2_(Co_1-x_Fe_x_)_17-y_Cr_y_, as well as the lattice parameters *a*, *c* and unit cell volume *V* at temperatures of 310 K and 660 K. (The Curie temperature of compound Nd_2_Co_17_ is derived from literature).^[1]^

| Samples  Nd_2_(Co_1-x_Fe_x_)_17-y_Cr_y_ | *T_C_* [K]  ($\pm$5 K) | *T*=310 K | | | *T*=660 K | | |
| --- | --- | --- | --- | --- | --- | --- | --- |
|  |  | *a* [Å] | *c* [Å] | *V* [Å] | *a* [Å] | *c* [Å] | *V* [Å] |
| *x* = 0.0, *y* = 0.0 | 1180 | 8.4271  ($\pm$0.17E-04) | 12.2487  ($\pm$0.29E-04) | 753.329  ($\pm$0.28E-02) | 8.4701  ($\pm$0.20E-04) | 12.2974  ($\pm$0.33E-04) | 764.070  ($\pm$0.33E-02) |
| *x* = 0.1, *y* = 4.7 (**S1**) | 442 | 8.4652  ($\pm$6.28E-05) | 12.2685  ($\pm$6.95E-05) | 761.387  ($\pm$1.48E-02) | 8.4998 | 12.3234 | 771.042 |
| *x* = 0.2, *y* = 4.3 (**S2**) | 514 | 8.4837  ($\pm$9.10E-05) | 12.3068  ($\pm$4.75E-05) | 767.080  ($\pm$0.91) | 8.5282  ($\pm$8.89E-05) | 12.3485  ($\pm$5.39E-05) | 777.788  ($\pm$0.67) |
| *x* = 0.3, *y* = 4.0 (**S3**) | 579 | 8.4813  ($\pm$5.00E-05) | 12.3290  ($\pm$7.00E-05) | 768.035 ($\pm$0.73E-02) | 8.5178  ($\pm$6.00E-05) | 12.3684  ($\pm$9.00E-05) | 777.136  ($\pm$0.91E-02) |
| *x* = 0.4, *y* = 3.7 (**S4**) | 601 | 8.4957 | 12.3705 | 773.260  ($\pm$1.02E-02) | 8.5562 | 12.3926 | 785.702  ($\pm$2.14E-02) |
| *x* = 0.5, *y* = 3.3 (**S5**) | 625 | 8.5101  ($\pm$7.78E-05) | 12.4068  ($\pm$1.07E-04) | 778.138 ($\pm$2.12E-02) | 8.5482  ($\pm$3.50E-04) | 12.4210  ($\pm$4.88E-04) | 786.019  ($\pm$9.48E-02) |
| *x* = 0.6, *y* = 3.0 (**S6**) | 612 | 8.5079  ($\pm$9.67E-05) | 12.4301  ($\pm$2.31E-04) | 779.207  ($\pm$1.35) | 8.5507  ($\pm$1.29E-04) | 12.4368  ($\pm$3.20E-04) | 787.491  ($\pm$1.12) |
| *x* = 0.7, *y* = 2.7 (**S7**) | 605 | 8.5311  ($\pm$8.08E-05) | 12.4652  ($\pm$5.02E-05) | 785.686  ($\pm$4.41E-02) | 8.5762  ($\pm$1.17E-04) | 12.4612  ($\pm$7.94E-05) | 793.690  ($\pm$8.42E-02) |
| *x* = 0.8, *y* = 2.3 (**S8**) | 562 | 8.5255  ($\pm$5.84E-04) | 12.4663  ($\pm$2.03E-04) | 783.638  ($\pm$0.66) | 8.5832  ($\pm$7.76E-04) | 12.4652  ($\pm$2.60E-04) | 794.636  ($\pm$0.49) |





**Figure S4.** The temperature dependence of (a) lattice parameter *a*, (b) lattice parameter *c*, (c) *c/a* ratio, and (d) unit cell volume *V* for the samples Nd_2_(Co_0.9_Fe_0.1_)_12.3_Cr_4.7_ (S1), Nd_2_(Co_0.6_Fe_0.4_)_13.3_Cr_3.7_ (S4), Nd_2_(Co_0.4_Fe_0.6_)_14_Cr_3_ (S6), Nd_2_(Co_0.3_Fe_0.7_)_14.3_Cr_2.7_ (S7), Nd_2_(Co_0.2_Fe_0.8_)_14.7_Cr_2.3_ (S8). The Curie temperatures are indicated by arrows.





**Figure S5.** (a)-(h) Temperature dependence of lattice parameters (*a_m_*, *c_m_*) and corresponding unit cell volume (*V_m_*) for Nd_2_(Co_1-x_Fe_x_)_17-y_Cr_y_ compounds. The red lines show the extrapolated paramagnetic lattice parameters (*a_p_*, *c_p_*) and unit cell volume (*V_p_*).





**Figure S6.** Temperature dependence of thermal expansion coefficients *α_a_*, *α_c_*, and *α_V_* for (a) Nd_2_(Co_0.5_Fe_0.5_)_13.7_Cr_3.3_ sample (S5), (b) Nd_2_(Co_0.4_Fe_0.6_)_14_Cr_3_ sample (S6), and (c) Nd_2_(Co_0.3_Fe_0.7_)_14.3_Cr_2.7_ sample (S7).





**Figure S7.** Temperature dependence of the 6c-6c nearest neighbor distance for Nd_2_(Co_0.2_Fe_0.8_)_14.7_Cr_2.3_ sample (S8).

**Table S2.** The numbers of neighbouring atoms, Wigner-Seitz cell volumes (WSC Volumes) and bond lengths in Nd_2_(Co_0.8_Fe_0.2_)_12.7_Cr_4.3_ at 310 K.

| Atom | Nd | Fe/Co/Cr | | | |
| --- | --- | --- | --- | --- | --- |
|  | 6c | 9d | 18f | 18h | 6c |
| Number of Nd neighbours | 1 | 2 | 2 | 3 | 1 |
| Number of Co neighbours | 19 | 10 | 11 | 9 | 13 |
| WSC-Volume [Å^3^] | 31.00(1) | 10.85(8) | 11.24(8) | 11.54(3) | 11.89(2) |
| Minimum bond length to neighbouring Co [Å] | 3.03(3) | 2.40(9) | 2.40(9) | 2.43(4) | 2.30(2) |
| Maximum bond length to neighbouring Co [Å] | 3.29(1) | 2.60(6) | 2.69(9) | 2.63(1) | 2.69(9) |
| Average bond length to neighbouring Co [Å] | 3.12(9) | 2.45(8) | 2.63(5) | 2.53(3) | 2.63(1) |





**Figure S8.** Temperature dependence of atomic positional parameters z_6c_, x_18f_, x_18h_, z_18h_, and z_6c_ for (a) Nd_2_(Co_0.9_Fe_0.1_)_12.3_Cr_4.7_ (S1), (b) Nd_2_(Co_0.4_Fe_0.6_)_14_Cr_3_ (S6), and (c) Nd_2_(Co_0.3_Fe_0.7_)_14.3_Cr_2.7_ (S7) samples: Nd atoms at the 6c Wyckoff position and Co/Fe/Cr atoms at the 18f, 18h, and 6c Wyckoff positions. The Curie temperatures are indicated by arrows.





**Figure S9.** Temperature dependence of average bonding distances between atoms at different Wyckoff positions and adjacent Co atoms, as well as Wigner-Seitz cell volumes, for: (a) Nd_2_(Co_0.8_Fe_0.2_)_12.7_Cr_4.3_ (S2), (b) Nd_2_(Co_0.4_Fe_0.6_)_14_Cr_3_ (S6), and (c) Nd_2_(Co_0.3_Fe_0.7_)_14.3_Cr_2.7_ (S7) samples, with Curie temperatures indicated by arrows.





**Figure S10.** (a) Magnetization versus field curves (*H* = 0 - 50000 Oe) for the Nd_2_(Co_0.8_Fe_0.2_)_12.7_Cr_4.3_ (S2) sample around the Curie temperature; (b) Arrott plots of *M^2^* versus *H/M* for the S2 sample; (c) Kouvel-Fisher plot for the spontaneous magnetization *M_S_(T)[dM_S_/dT]^-1^* (left scale) versus temperature *T* and the inverse initial susceptibility *χ^-1^[dχ^-1^/dT]^-1^* (right scale) versus temperature *T*; (d) Modified Arrott plots of *M^1/β^* as a function of *(H/M)^1/γ^* leading to the values *γ* = 1.04 and *β* = 0.60; (e) Scaling plots of *M|ε|^-β^* versus *H|ε|^-(β+γ)^* (using *β* and *γ* as discussed in the text; cf. Equation(9)), indicating the universal behaviour of the curves below and above *T_C_* for the S2 sample; (f) Critical isotherms of magnetization as a function of the magnetic field close to the Curie temperature for the S2 sample - the inset shows the data plotted on a log *M* versus log *H* graph and the linear fit obtained at the critical transition temperature.


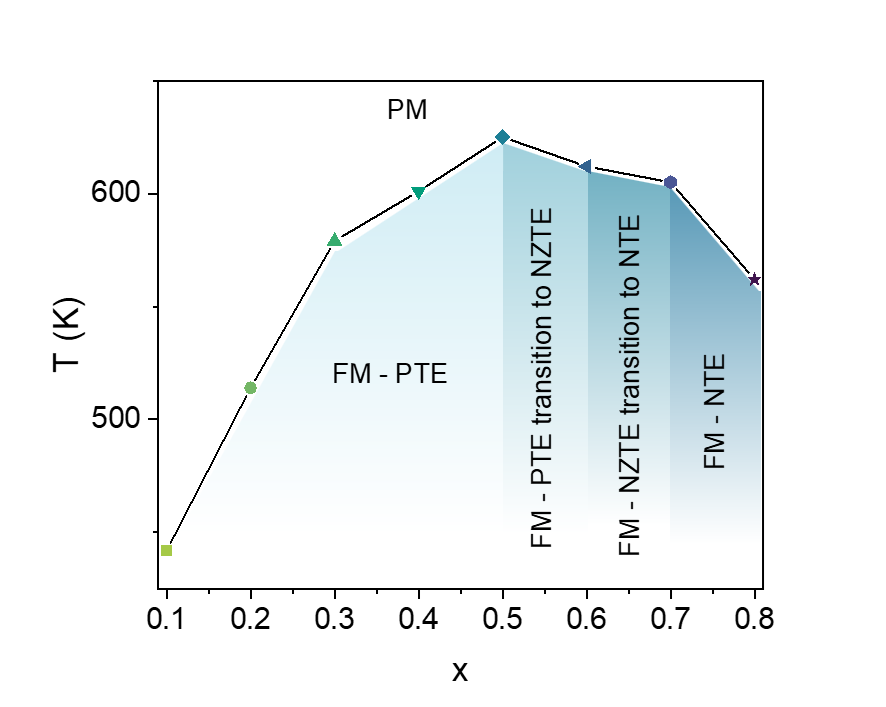


**Figure S11.** Schematic phase diagram of the Nd_2_(Co_1-x_Fe_x_)_17-y_Cr_y_ system (*x*-axis: Fe content x; *y*-axis: Curie temperature *T_C_*), showing the non-monotonic variation and corresponding positive-to-negative thermal expansion transition with increasing Fe content for different Cr contents (y).

**Table S3.** Summary of surface scan morphology map, total elemental distribution map of surface scan, elemental distribution submaps of surface scan, EDS mapping of surface scan and the quantitative elemental content data for Nd_2_(Co_0.3_Fe_0.7_)_14.3_Cr_2.7_ (S7) sample.

| **Sample Description** | Nd_2_(Co_0.3_Fe_0.7_)_14.3_Cr_2.7_ (S7) |
| --- | --- |
| Surface scan morphology map | |
| 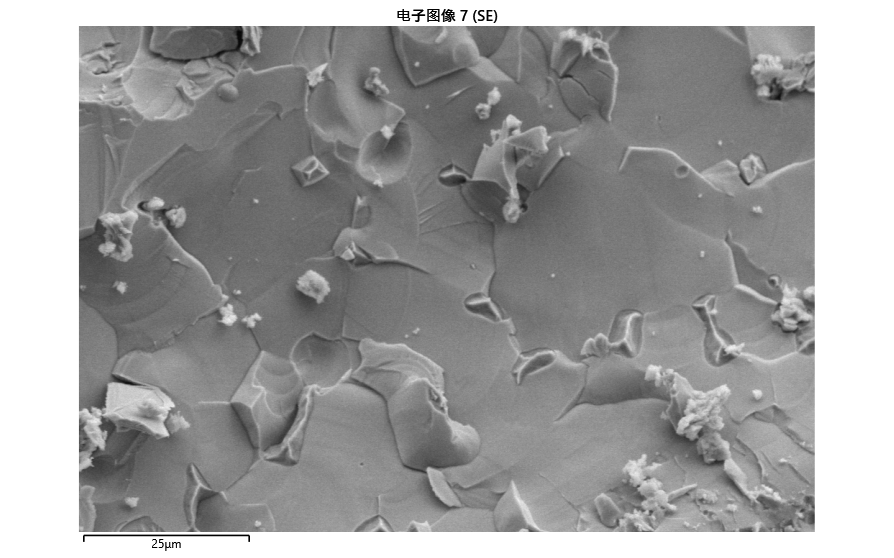 | |
| Total elemental distribution map of surface scan | |
| 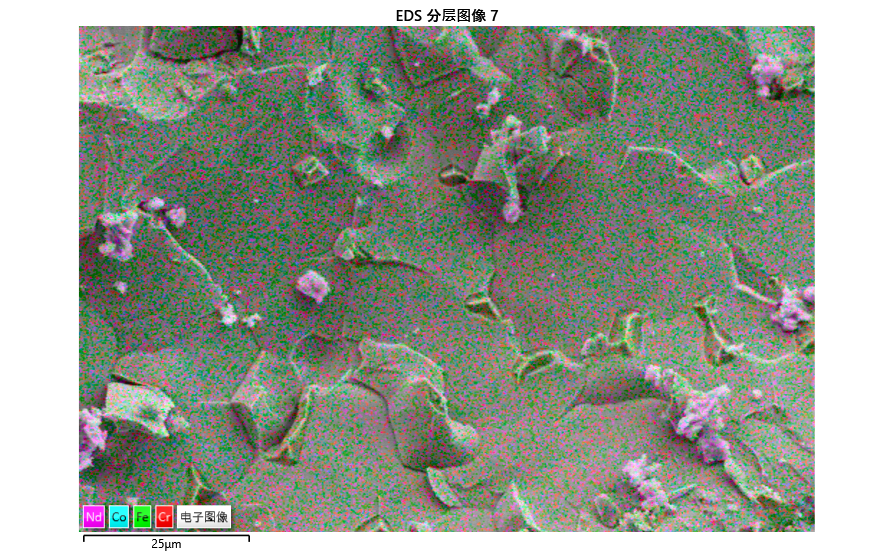 | |
| Elemental distribution submap of surface scan | |
| 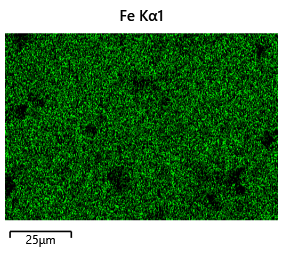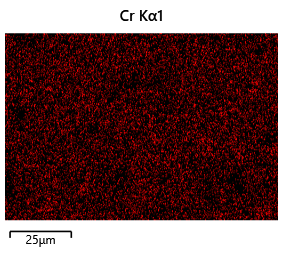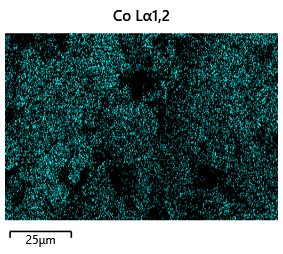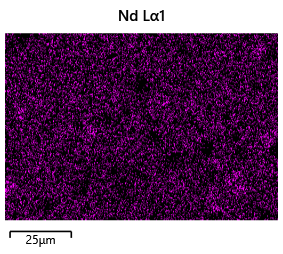 | |
| EDS mapping of surface scan | |
| 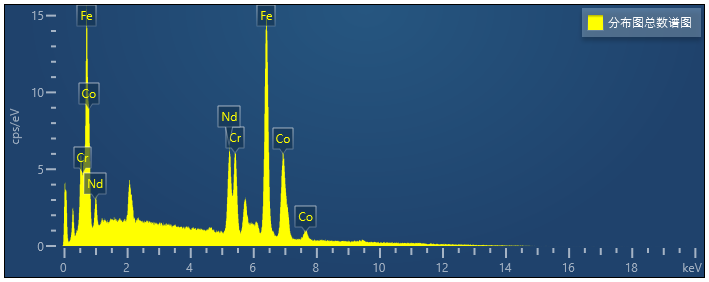 | |
| EDS table of surface scan | |
| \| **Element** \| **Line type** \| **Wt%** \| **Wt% Sigma** \| **At%** \| \| --- \| --- \| --- \| --- \| --- \| \| Cr \| K series \| 9.50 \| 0.15 \| 12.13 \| \| Fe \| K series \| 44.32 \| 0.27 \| 52.71 \| \| Co \| K series \| 20.86 \| 0.25 \| 23.51 \| \| Nd \| L series \| 25.32 \| 0.28 \| 11.66 \| \| **Total amount** \|  \| 100.00 \|  \| 100.00 \| | |

**Table S4.** Summary of surface scan morphology map, total elemental distribution map of surface scan, elemental distribution submaps of surface scan, EDS mapping of surface scan and the quantitative elemental content data for Nd_2_(Co_0.2_Fe_0.8_)_14.7_Cr_2.3_ (S8) sample.

| **Sample Description** | Nd_2_(Co_0.2_Fe_0.8_)_14.7_Cr_2.3_ (S8) |
| --- | --- |
| Surface scan morphology map | |
| 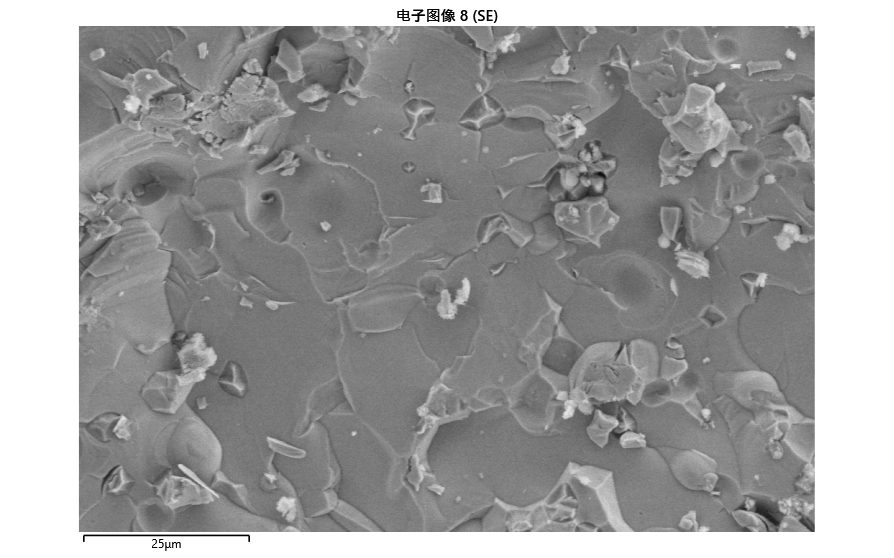 | |
| Total elemental distribution map of surface scan | |
| 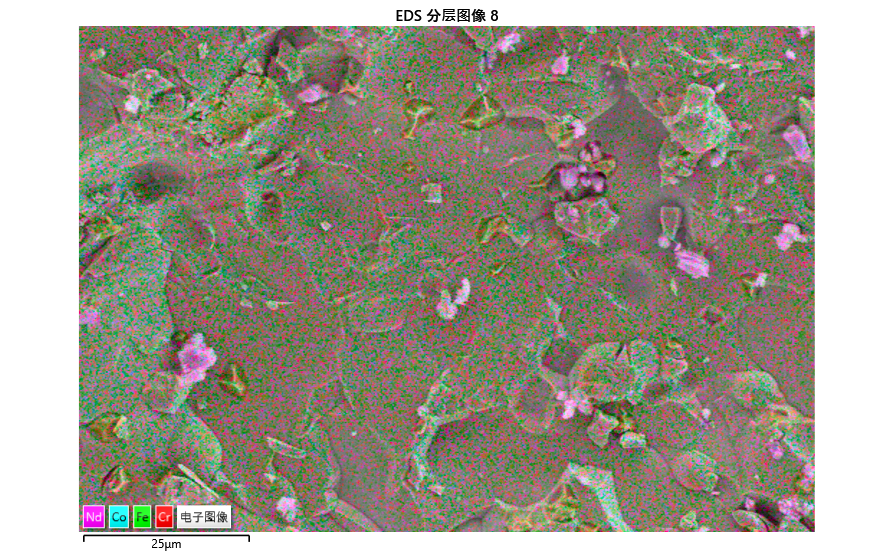 | |
| Elemental distribution submap of surface scan | |
| 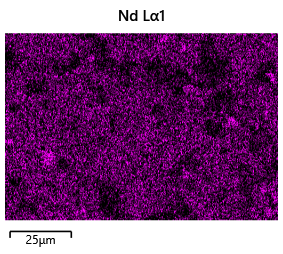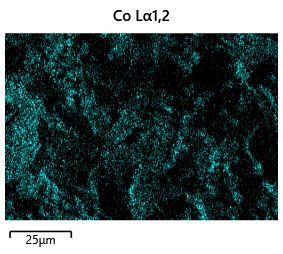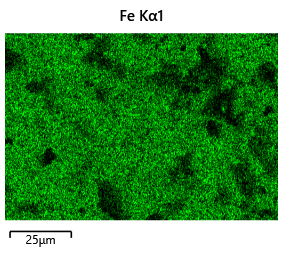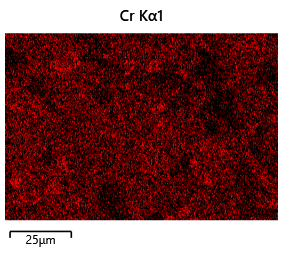 | |
| EDS mapping of surface scan | |
| 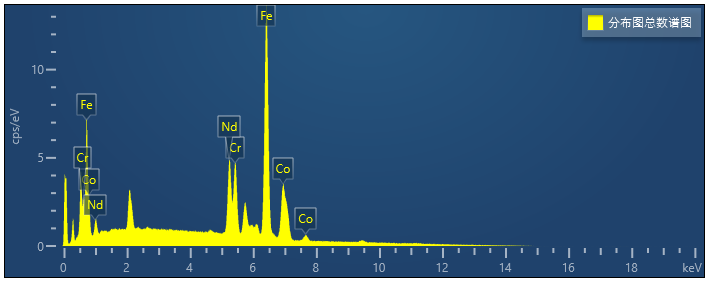 | |
| EDS table of surface scan | |
| \| **Element** \| **Line type** \| **Wt%** \| **Wt% Sigma** \| **At%** \| \| --- \| --- \| --- \| --- \| --- \| \| Cr \| K series \| 9.18 \| 0.10 \| 11.66 \| \| Fe \| K series \| 51.47 \| 0.19 \| 60.85 \| \| Co \| K series \| 14.30 \| 0.15 \| 16.03 \| \| Nd \| L series \| 25.04 \| 0.19 \| 11.46 \| \| **Total amount** \|  \| 100.00 \|  \| 100.00 \| | |

**Table S5.** Summary of EDS Spectrum and the quantitative result for Nd_2_Co_17_ sample.

| **Sample Description** | Nd_2_Co_17_ |
| --- | --- |
| EDS Spectrum | |
| 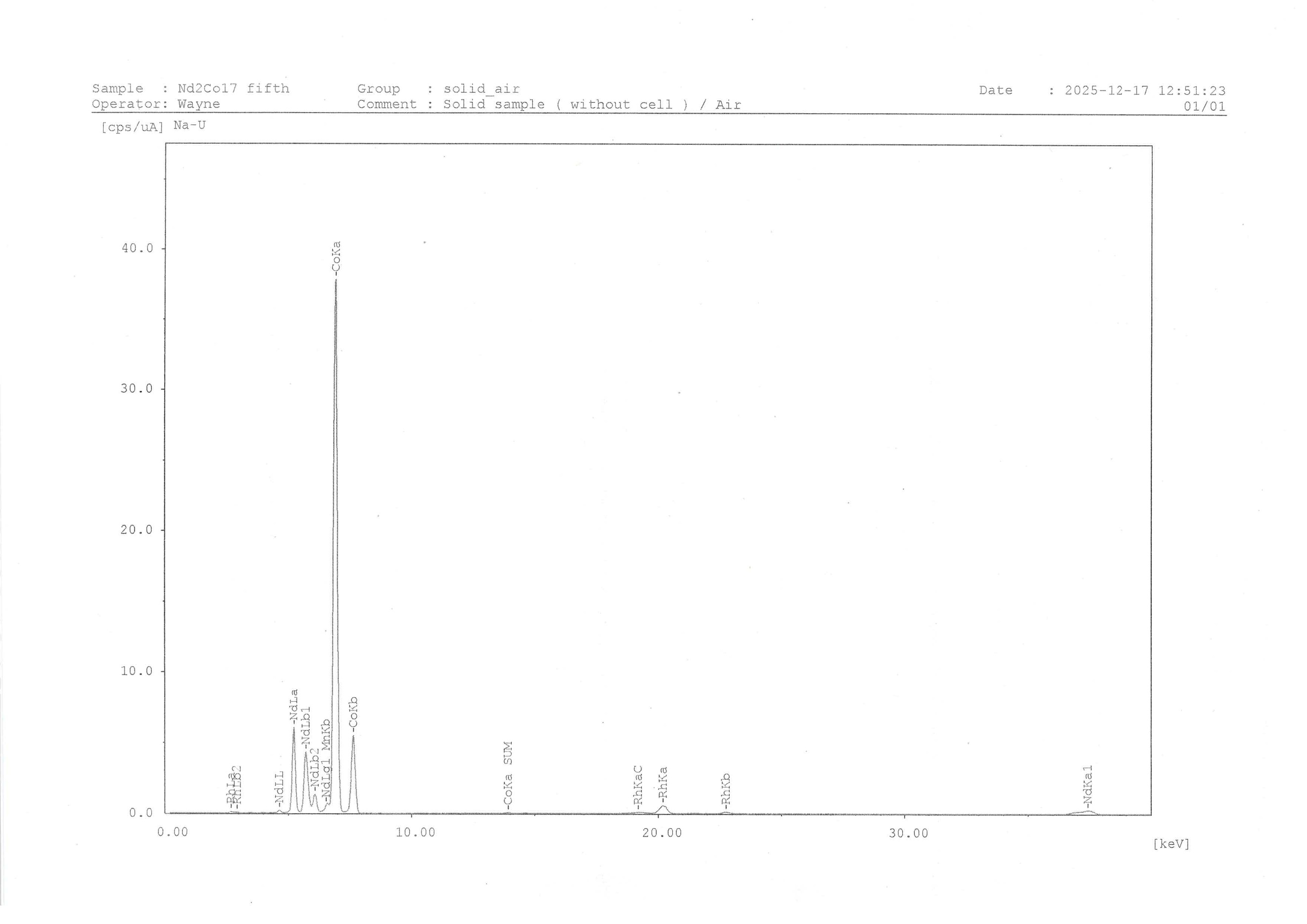 | |
| Quantitative Result | |
| \| **Element** \| **Line** \| **Wt%** \| **At%** \| \| --- \| --- \| --- \| --- \| \| Co \| CoKα \| 80.476 \| 90.90 \| \| Nd \| NdKα \| 19.524 \| 9.10 \| \| **Total amount** \|  \| 100.00 \| 100.00 \| | |

**Reference**

^[1]^ L. Zhang, Y. N. Liang, D. C. Zeng, J. C. P. Klaasse, E. Brück, Z. Y. Liu, F. R. de Boer, and K. H. J. Buschow, Physica B: Condensed Matter **291** (1), 117 (2000).
